# Uncommon cause of large bowel obstruction in a pediatric patient: A case of phytobezoar induced by peas

**DOI:** 10.1016/j.radcr.2025.01.097

**Published:** 2025-03-08

**Authors:** Kinfemicheal Tilahu Yigzaw, Dawit Aysheshim Mulualem, Bethlehem Aliye Asfaw, Melkamu Temesgen Moges, Biniyam Alebachew Tegegne, Meseret Hussien Shibesh, Mesfin Tesera Wassie

**Affiliations:** a School of Medicine, College of Medicine and Health Sciences, Comprehensive Specialized Hospital, University of Gondar, Gondar, Ethiopia; b Department of Surgery, College of Medicine and Health Sciences, Comprehensive Specialized Hospital, University of Gondar, Gondar, Ethiopia; c Department of Nursing, College of Medicine and Health Sciences, Comprehensive Specialized Hospital, University of Gondar, Gondar, Ethiopia; d Department of Pediatric Surgery, College of Medicine and Health Sciences, Comprehensive Specialized Hospital, University of Gondar, Gondar, Ethiopia

**Keywords:** Peas, Pediatric, Large Bowel, Bezoars, Intestinal obstruction, Case reports

## Abstract

Phytobezoars, which are indigestible plant materials located in the gastrointestinal tract, are an uncommon cause of intestinal blockage, especially in pediatric patients. This report details a case involving a 2-year-old girl who experienced a large bowel obstruction caused by phytobezoars resulting from excessive consumption of peas who exhibited symptoms of abdominal swelling, an inability to pass stool and gas, and vomiting, abdominal distension with visible peristalsis with a plain abdominal X-ray finding of multiple air-fluid levels and enlarged bowel loops. Surgical intervention was initiated due to a suspected small bowel obstruction and reveals impacted phytobezoars and hard fecal matter, necessitating a colotomy for their removal. There is no report of case causing large bowel obstruction due to peas and up to our best, it is the first case to be documented of such a large obstruction resulting from peas. This case highlights the significance of considering phytobezoars in young patients who present with risk factors and gastrointestinal symptoms, particularly following dietary changes. Timely identification and suitable management can avert complications and unnecessary surgical interventions.

## Introduction

Phytobezoars are indigestible plant substances that may lead to gastrointestinal obstruction, especially among pediatric patients [1]. They account for 0.4%-4% of all gastrointestinal blockages and are the most prevalent form of bezoar [1,2]. Risk factors include poor dental hygiene, a history of surgery, and diets high in fiber, particularly those containing foods like peas and beans [3].

Common symptoms include abdominal bloating, nausea, and an inability to pass stool or gas [4]. Since imaging techniques such as X-rays and CT^1^ scans have restricted sensitivity, diagnosis usually takes place only when surgery is performed [5,6].

This report discusses a case involving a 2-year-old girl who experienced a significant bowel obstruction caused by phytobezoars resulting from excessive consumption of peas. The case highlights the necessity of recognizing phytobezoars in pediatric patients, particularly after alterations in their diet, and underscores the significance of prompt diagnosis and treatment.

## Case presentation

A 2-year-old toddler female initially presented with a complaint of abdominal distension, failure to pass faces and flatus, and multiple episodes of vomiting of ingested matter of 1 day duration. A day before she ate a spike of peas much in amount with her friends. Otherwise, she has no history of recent Surgery or previous history of surgery, no self or family history of known chronic medical illness like diabetes mellitus, heart disease and others. She also has no similar illness in the family. She is from a family size of 5 peoples with adequate monthly income, and whose work is farming.

Upon examination, she was tachycardic (134 bpm^2^), and tachypneic (40 bpm^3^), and had signs of dehydration were there. She has an incomplete tooth eruption. The abdomen was distended with visible peristalsis, with impacted stool findings per rectal examination.

**Fig. 1:**
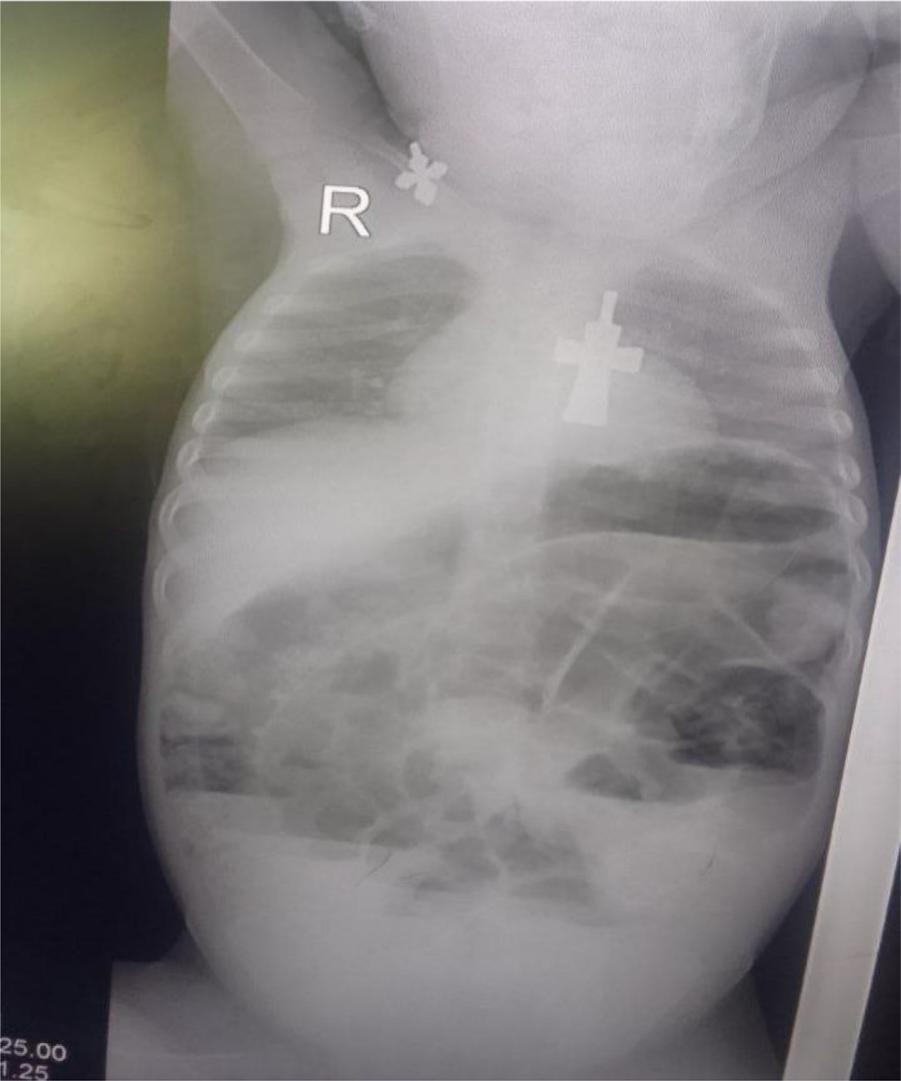
Plain abdominal X-ray shows multiple air-fluid levels with dilated bowel loops and absent rectal air shadow.

Her serum chemistry panel was in the normal range including renal function test of Creatinine of 0.42 mg/dl^4^, blood urea nitrogen of 9, serum potassium, and sodium chlorine of 4.4 mEq/L^5^, 141 mEq/L, 101 mEq/L respectively. The complete blood count profiles also in normal range (WBC^6^: 9,000/µL^7^, HGB^8^: 12.9 g/dL^9^ and platelets: 224,000/µL). A plain abdominal X-ray shows multiple air-fluid levels with dilated bowel loops and absent rectal air shadow (Fig. 1).

**Fig. 2:**
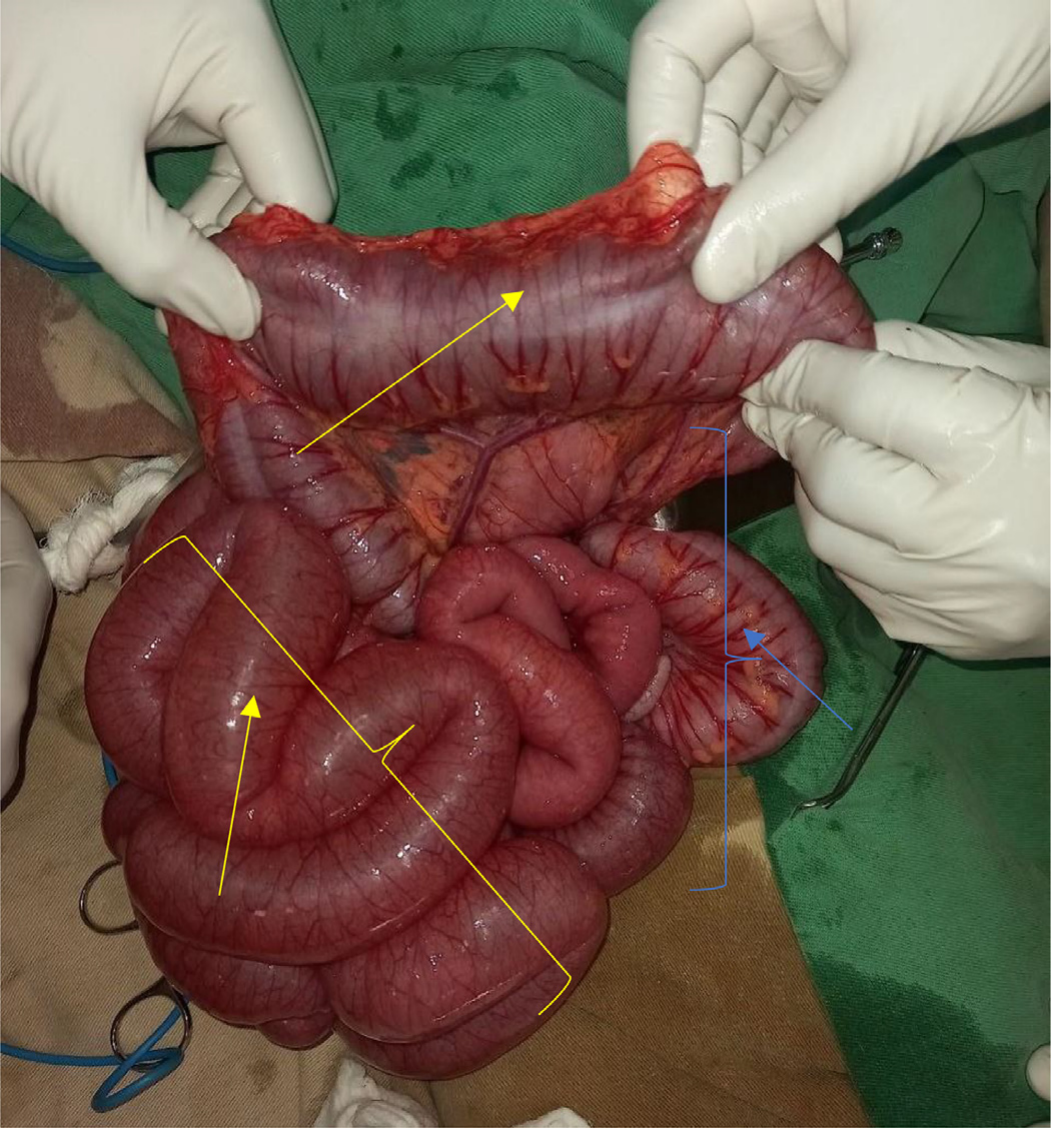
The entire small bowel to the transverse colon was distended (yellow arrows). Distally, the descending and the sigmoid colon collapsed (blue arrows).

**Fig. 3:**
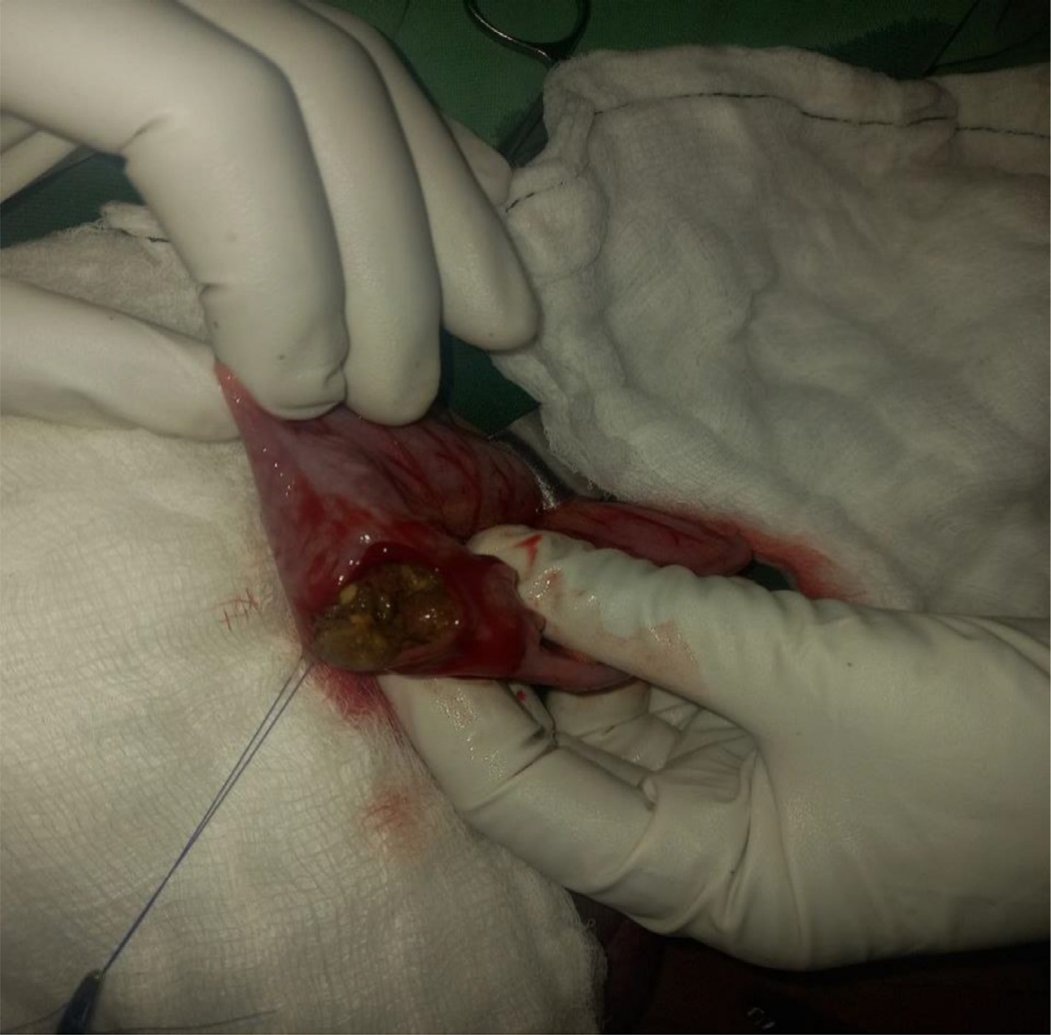
Opened sigmoid dome showing impacted stool with peas.

**Fig. 4:**
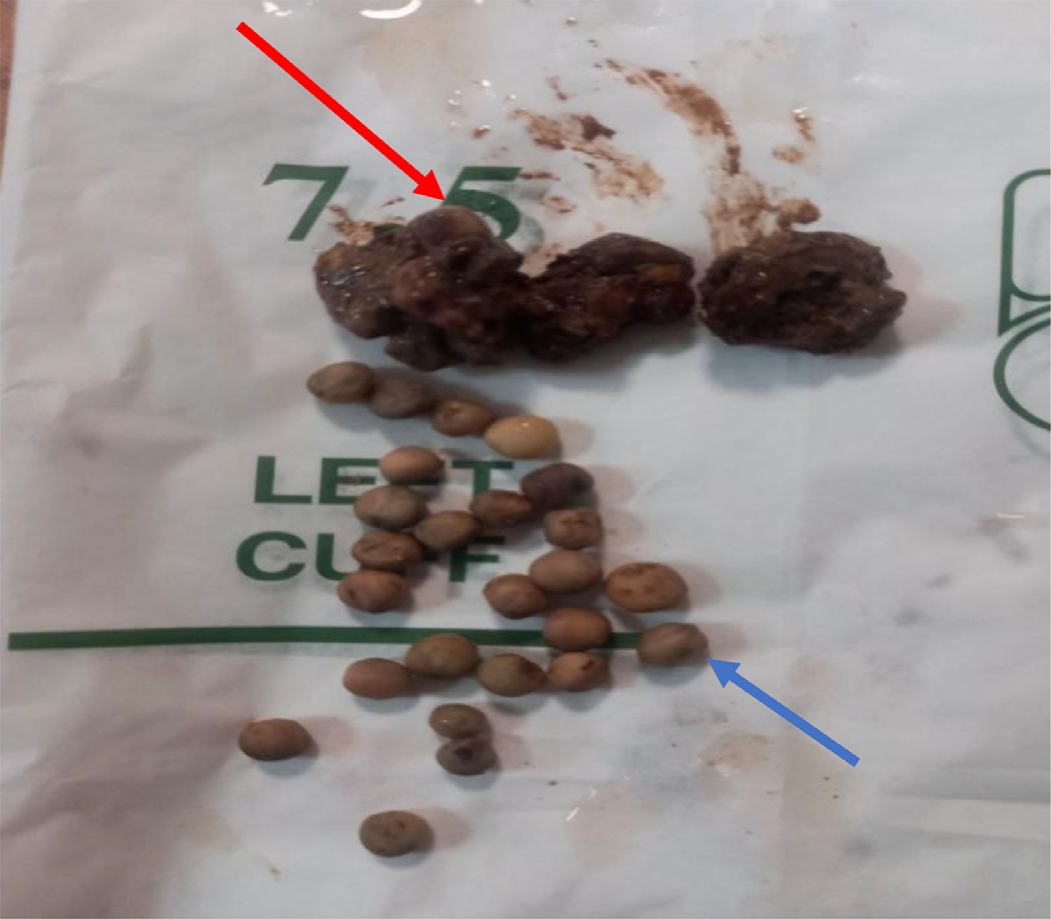
Showing a sample of multiple peas (blue arrow) with hard fecal matter (red arrow).

The patient was resuscitated with Normal saline, Nasogastric tube was inserted for the decompression purpose. Without delay, the operation was performed for the impression of small bowel obstruction. Intraoperatively, the entire small bowel to the transverse colon was distended, distally descending, and the sigmoid colon collapsed (Fig. 2). Multiple obstructive intraluminal masses suspicious for fecaloma or tumor which starting from splenic flexure to the rectum, sparsely found on the descending colon and rectum but more impacted on the sigmoid colon. An effort was made to fragment the material digitally and guide it toward the rectum; however, the mass was quite immobile at the sigmoid dome. Colotomy was done at the sigmoid dome (Fig. 3). Phytobezoars (peas) with hard fecal matter were identified upon opening the colon (Fig. 4). Distal and proximal bezoars were removed with milking and distally irrigated until no pass via the rectum. Finally, the colotomy was closed.

**Fig. 5:**
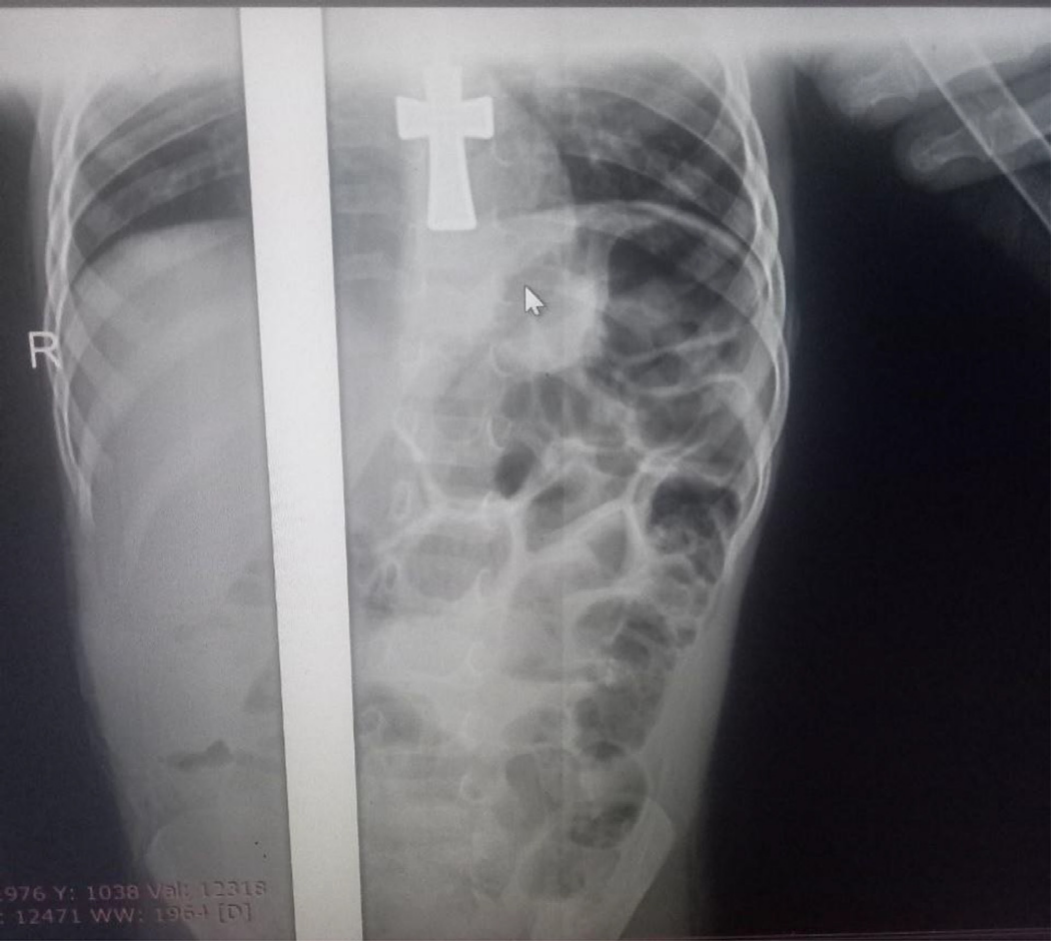
Postoperative X-ray reveals unremarkable finding with no air fluid level or distended bowel loop.

Postoperatively, the patient was put on NPO^10^, broad-spectrum antibiotics, wound care, and antipain. Her post operation condition was smooth with no post operation complication. After 1 week of stay in the hospital, the patient was discharged improved with a short appointment. After 1 week of the appointment, she was in good condition passing stool and tolerating feeding. Her post operation plain abdominal X-ray also reveals unremarkable finding (Fig. 5).

After 1 month of appointment, there was uneventful course.

## Discussion

Phytobezoars are indigestible plant materials located within the gastrointestinal tract [1]. The term "bezoar" originates from the Farsi word "padzahr," which translates to "antidote" or "antitoxin" [7].

There are many other types of bezoars involved in intestinal obstruction such as trichobezoar (hair bezoar), pharmacobezoars, diospyrobezoar (persimmon bezoar), and lactobezoar [8]. Although uncommon, bezoars represent the most frequently encountered foreign material in the gastrointestinal system. The prevalent type of bezoar is the phytobezoar [1].

Phytobezoars are infrequent and represent just 0.4%-4% of all instances of gastrointestinal blockage, primarily occurring in the stomach or small intestine, but Intestinal blockages resulting from colonic bezoars are very uncommon [2].

The primary contributing factors for phytobezoars include previous abdominal surgical procedures and inadequate dental health [9]. Other less common risk factors for bezoars are frequent use of antacids, opiate use, diabetic mellitus, tumor, and diverticula, and increased intake of fruit and vegetables like beans, peas, carrots, and others [3,7]. Our patient has incomplete dental eruption and ate a large amount of peas a day before admission, which is one of the risk factors for phytobezoars in this age group.

The mechanisms behind the formation of bezoars can be categorized into mechanical and chemical factors [7]. Dysfunction or removal of pyloric function due to various procedures permits inadequately hydrolyzed food particles to enter the small intestine, leading to an increase in the formation of phytobezoars [6]. Gastrointestinal immotility, which includes conditions like gastric stasis or delayed gastric emptying, leads to an extended presence of material in the intestines, thereby facilitating the development of bezoars [10]. Reduced stomach acidity, as seen with truncal vagotomy, hampers the breakdown of consumed food, resulting in a higher volume of undigested food material reaching the distal intestine [10].

Phytobezoars do not exhibit distinct signs or symptoms when they occur like in our patient who presented with nonspecific sign and symptoms and X-ray showed small bowel obstruction. The majority of intestinal bezoars manifest as a complete blockage of the intestines [4]. They frequently show symptoms of crampy abdominal discomfort accompanied by vomiting [7]. A 2008 study of 15 instances of small bowel obstruction caused by phytobezoars found that 13 of the 15 patients showed no fever and experienced no peritonitis, even though nasogastric drainage indicated the presence of bilious fluid. Only 2 cases manifested as acute abdominal conditions that necessitated emergency surgery [5].

It is unusual to diagnose a phytobezoar before surgery; however, when identification is achieved, effective medical treatment can be administered [6]. Phytobezoars should be considered in patients with a history of gastric or abdominal surgery, significantly poor dental health, or who have recently increased their fiber intake before seeking treatment [9]. In cases where phytobezoars are suspected, it has been customary to conduct a physical examination and an abdominal X-ray; however, these diagnostic methods have a sensitivity of only 10% [5]. CT continues to be the most effective technique for identifying a phytobezoar, including colonic bezoar [11]. It has a positive predictive value of merely 20%, showcasing a stool-like mass featuring a solid border and a heterogeneous, “mottled gas” center that does not absorb iodine contrast, situated between proximal distended segments of bowel and distal collapsed sections of bowel [5].

The bowel typically does not experience ischemia unless discovered at a very advanced stage. A comprehensive evaluation of the intestines and stomach is essential during surgical procedures, as the presence of concurrent bezoars is quite frequent [6,7].

Our patient presented with typical bowel obstruction signs and symptoms and plain abdominal radiography suggestive of small bowel obstruction.

Phytobezoars can be treated through various surgical and medical approaches that both demonstrate a positive outlook [4,5]. If a phytobezoar is identified before surgery, it is probable that medical interventions will be effective [12].

Various medical treatments have been tried successfully to eliminate phytobezoars, such as Coca-Cola, Adolph's Meat Tenderizer, L-cysteine, cellulase, cellulase combined with metoclopramide, papain, water jet, pineapple juice, normal saline, 0.1 M hydrochloric acid, sodium bicarbonate, pancrelipase, pancreatin, and 12% zinc chloride [7].

Surgical intervention should include maneuvering the phytobezoar towards the distal large intestine. An enterotomy or colotomy is necessary if the bezoar cannot be broken down and moved distally. Ultimately, a resection is warranted in situations involving intestinal necrosis, if the bezoar does not separate from the intestinal lining, or if there is a high likelihood of the phytobezoar reappearing [7]. In our case, it was difficulty of milk the mass distally. For this colotomy was done.

The case discussed here is notable because large bowel obstructions due to bezoars are exceedingly uncommon. To the best of our knowledge, there are only 2 report of a large bowel obstruction caused by a bezoar. Furthermore, there is no report of case causing large bowel obstruction due to peas. It is the first instance documented of such a large obstruction resulting from peas.

Since 20% of patients experienced recurrent bezoars, it may be helpful to steer clear of foods that trigger them, along with ensuring proper chewing and sufficient hydration [2].

## Conclusion

Phytobezoars, though uncommon, should be considered in situations where multiple minor risk factors for their development are present, including minor modifications in diet that lead to a higher intake of vegetable fiber. A strong level of suspicion should also be maintained for patients with risk factors and characteristic CT scan findings for these uncommon conditions to prevent unnecessary surgical procedures.

## Ethical approval

Not applicable.

## Author contributions

**KTY:** Writing—original draft, Conceptualization. **DAM:** Writing—original draft, patient care. **MTM:** Writing—review and editing, Resources. **BAA:** Writing—review and editing. **BAT:** Writing—review and editing. **MHS:** Writing—original draft, patient care. **MTW:** Writing—review and editing, patient care, Supervision.

## Patient consent

Informed consent was obtained from the mother of the patient and a copy of the written consent is available for review by editor in chief of this journal.

## Footnotes

1 CT, computed tomography.

2 BPM, beat per minute.

3 Bpm, breath per minute.

4 Mg/dl, milligram per deciliters.

5 mEq/L, milliequivalent per liters.

6 WBC, white blood cells.

7 µL, microliters.

8 HGB, hemoglobin.

9 g/dL, gram per deciliters.

10 NPO, nil per os.
